# Animal models to investigate the effects of inflammation on remyelination in multiple sclerosis

**DOI:** 10.3389/fnmol.2022.995477

**Published:** 2022-11-03

**Authors:** Marjan Gharagozloo, Jackson W. Mace, Peter A. Calabresi

**Affiliations:** ^1^Department of Neurology, Johns Hopkins University School of Medicine, Baltimore, MD, United States; ^2^Solomon H. Snyder Department of Neuroscience, Johns Hopkins University, Baltimore, MD, United States

**Keywords:** multiple sclerosis, remyelination, inflammation, cuprizone, EAE, adoptive transfer

## Abstract

Multiple sclerosis (MS) is a chronic inflammatory, demyelinating, and neurodegenerative disease of the central nervous system (CNS). In people with MS, impaired remyelination and axonal loss lead to debilitating long-term neurologic deficits. Current MS disease-modifying drugs mainly target peripheral immune cells and have demonstrated little efficacy for neuroprotection or promoting repair. To elucidate the pathological mechanisms and test therapeutic interventions, multiple animal models have been developed to recapitulate specific aspects of MS pathology, particularly the acute inflammatory stage. However, there are few animal models that facilitate the study of remyelination in the presence of inflammation, and none fully replicate the biology of chronic demyelination in MS. In this review, we describe the animal models that have provided insight into the mechanisms underlying demyelination, myelin repair, and potential therapeutic targets for remyelination. We highlight the limitations of studying remyelination in toxin-based demyelination models and discuss the combinatorial models that recapitulate the inflammatory microenvironment, which is now recognized to be a major inhibitor of remyelination mechanisms. These models may be useful in identifying novel therapeutics that promote CNS remyelination in inflammatory diseases such as MS.

## Introduction

Multiple sclerosis (MS) is a chronic disease of the central nervous system (CNS), characterized by peripheral immune infiltration, demyelination, reactive gliosis, and axonal degeneration (Reich et al., 2018). Multiple disease-modifying drugs have been effective in the treatment of the inflammatory stage of the disease and demonstrated a significant reduction in the formation of new lesions and annualized relapse rate (Bierhansl et al., 2022). However, none of these drugs meaningfully slow the accumulation of disability in progressive MS cases in which there are no superimposed relapses or new MRI lesions. Chronic demyelination renders axons susceptible to neurodegeneration through loss of trophic support, and remyelination is neuroprotective and possibly could restore function. While numerous potential remyelinating compounds have been identified in preclinical research, they have shown limited success in MS clinical trials (Gharagozloo et al., 2022). In MS, chronic inflammation and axonal pathology interfere with remyelination. Both intrinsic defects in oligodendrocyte lineage cells and extrinsic inhibitory cues in the lesion microenvironment negatively affect oligodendrocyte progenitor cells (OPC) differentiation and the generation of new myelin sheaths (Gharagozloo et al., 2022). Nevertheless, the use of animal models is essential for understanding the complex cellular and molecular mechanisms underlying these processes and for developing successful remyelination therapies for MS.

Over the past several decades, various animal models have been used to test novel therapies for MS and study the immunopathological mechanisms. The most commonly used animal model of MS is experimental autoimmune encephalomyelitis (EAE), in which neuroinflammation is the predominant feature observed after immunization with myelin-derived antigens. EAE reflects important aspects of MS immunopathology and has directly led to the development of several effective approved therapies for MS. However, the utility of this model is limited for understanding the remyelination process and identifying strategies for myelin repair due to the unpredictable spinal cord locations of lesions, and the extensive axonal pathology that occurs in this model. Toxin-based models such as cuprizone are commonly used to study demyelination and remyelination in the CNS, but they mostly lack the chronic inflammation that is known to exist in MS plaques. To better recapitulate the complexity of MS pathology, several mouse models have been developed that combine an immune response with toxin-induced demyelination. These combinational models may be useful to identify the molecular factors contributing to remyelination failure and to test novel therapies that promote myelin repair and prevent MS progression.

**FIGURE 1:**
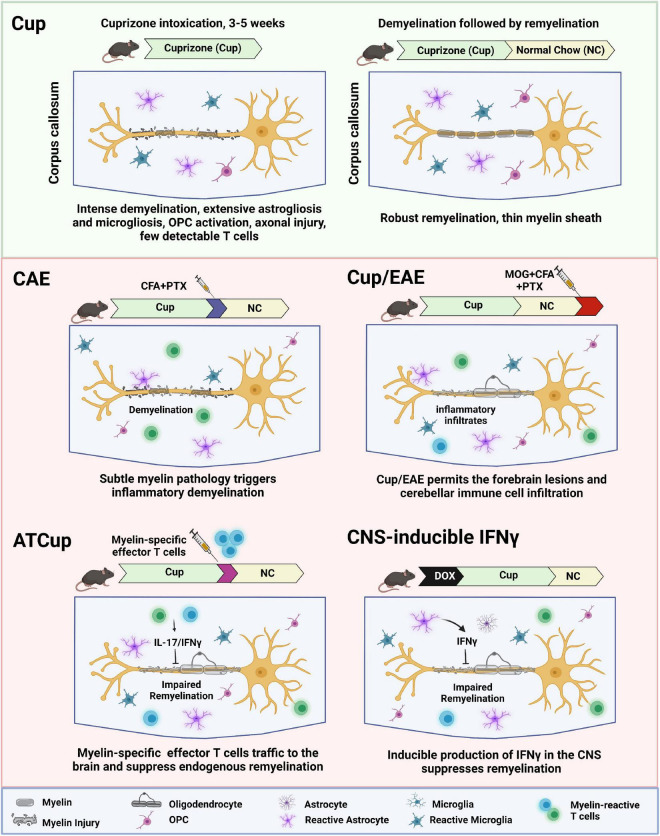
Schematic overview of inflammatory demyelination models, showing how inflammation contributes to de- and re-myelination following cuprizone intoxication. ATCup, Adoptive Transfer Cuprizone; CAE, Cuprizone Autoimmune Encephalitis; CFA, Complete Freund’s Adjuvant; DOX, Doxycyclin; EAE, Experimental Autoimmune Encephalomyelitis; MOG, Myelin oligodendrocyte glycoprotein; OPC, Oligodendrocyte precursor cells; PTX, Pertussis toxin.

In this review, we describe the current animal models that have provided insight into the mechanisms by which demyelination and impaired remyelination occur in MS. We highlight the complexities and challenges of studying remyelination, while discussing future perspectives of using animal models to develop novel remyelination therapies for MS. We summarize the current de- and re-myelination models in Table 1 and further discuss the combinational inflammatory models in Figure 1.

**TABLE 1:** Mouse models for studying inflammatory-mediated de- and re-myelination in MS.

Model	Induction	Study design	Main findings	References
Toxin-induced demyelination	Acute cuprizone model	Cuprizone chow, 4–6W	Robust white matter demyelination, few detectable CD4 or CD8 cells in the brain, intact BBB	Matsushima and Morell, 2001; Hiremath et al., 2008; Sen et al., 2019
	Acute Cuprizone model	Cuprizone chow, 4-6 W	Increased BBB permeability precedes demyelination in the cuprizone model	Berghoff et al., 2017; Shelestak et al., 2020
	Acute Cuprizone model	Cuprizone, 3W→ Normal chow, 2W	Primary oligodendrocyte apoptosis, followed by the formation of degenerative forebrain lesions	Scheld et al., 2016
	Acute and Chronic Cuprizone	Cuprizone chow 3, 5, and 11W	Cuprizone intoxication triggers secondary recruitment of T lymphocytes, particularly CD8 + cytotoxic T cells	Kaddatz et al., 2021
	Chronic Cuprizone	Cuprizone chow, 12W	Significant reduction of peripheral T cells as well as splenic atrophy, no T cells were detected in the brain	Sen et al., 2019
	Diphtheria toxin (DTA model)	Tamoxifen-dependent oligodendrocyte ablation in Plp1-CreERT;ROSA26-eGFP	Oligodendrocyte loss and demyelination are not sufficient to cause T cells activation and CNS infiltration; No BBB disruption or lymphocyte infiltration to the CNS	Traka et al., 2010; Pohl et al., 2011
	Diphtheria toxin (DTA model)	Tamoxifen-dependent Plp1-CreERT; ROSA26-eGFP (Long term, 30W)	T cell infiltration into the CNS of during the late-onset disease, increased numbers of T lymphocytes in the CNS and MOG-specific T cells in lymphoid organs	Traka et al., 2016
	Diphtheria toxin (DTR model)	Double transgenic animals express the DTR on the surface of oligodendrocytes treated with DTX	Toxin-induced oligodendrocytes death; No BBB disruption or anti-CNS autoimmunity was observed	Locatelli et al., 2012

Toxin + Immunostimulators	Cuprizone + CFA + PTX (CAE model)	Cuprizone 2W→ Immunization with CFA→ Normal chow 2W	Subtle myelin pathology triggers inflammatory demyelination	Caprariello et al., 2018
	Cuprizone + PTX	Cuprizone chow, 5, 12W→ PTX, 3 doses (400 ng on days 14, 16, and 23)	Cuprizone causes splenic atrophy and immunosuppression; No T cells were detected in the brain even in the presence of PTX	Sen et al., 2019

Toxin + Active Autoimmunity	Cuprizone + EAE (MOG)	Cuprizone, 4W→ Normal chow, 2W→ Immunization with MOG35–55	Cuprizone treatment protects mice against the development of EAE	Maña et al., 2009
	Cuprizone + EAE (PLP)	Immunization with PLP 139-151→ Cuprizone started after recovery from the first EAE episode (PID22)	Cuprizone treatment improves relapsing-remitting EAE in SJL mice	Maña et al., 2009
	Cuprizone + EAE (TMEV)	TMEV infection→ Cuprizone, PID 35-70→ Normal Chow until PID196	Cuprizone impedes progression of TMEV-induced demyelination	Herder et al., 2012
	Cuprizone + EAE (PLP)	Cuprizone 1W→ Immunization with PLP Immunization with PLP, 1W→ Cuprizone	Cuprizone treatment decreases the severity of EAE in the SJL mouse	Emerson et al., 2001
	Cuprizone + EAE (MOG) (Cup/EAE)	Cuprizone, 3W→ Normal chow, 2W→ Immunization with MOG35–55	Cup/EAE induces cerebellar immune cell infiltration and causes forebrain lesions	Scheld et al., 2016; Rüther et al., 2017
		Cuprizone, 3W→ Normal chow, 4W→ Immunization with MOG35–55		
	Diphtheria toxin + EAE (MOG)	DTR mice and controls were treated with DTX and immunized with MOG35–55.	Absence of CNS inflammation after oligodendrocyte death is not a result of T cell tolerance	Locatelli et al., 2012

Toxin + Passive Autoimmunity	Cuprizone + adoptive transfer (ATCup)	Cuprizone 4W→ Th17 adoptive transfer→ Normal chow 2W	IL-17/IFNγ cells suppress endogenous remyelination by targeting OPCs	Baxi et al., 2015; Kirby et al., 2019

Genetic model of inflammatory CNS	Cuprizone in *GFAP/tTA*;*TRE/IFN*γ mice ± DOX	Cuprizone 6W→ Normal chow 3W	IFNγ expression in the CNS significantly suppresses remyelination, promotes CD8 infiltration, and MHC I expression on OPCs	Lin et al., 2006; Kirby et al., 2019

ATCup, adoptive transfer cuprizone; BBB, blood–brain barrier; CAE, cuprizone autoimmune encephalitis; CFA, complete Freund’s adjuvant; DOX, doxycycline; DTA, diphtheria toxin A; DTR, diphtheria toxin receptor; EAE, experimental autoimmune encephalomyelitis; MOG, myelin oligodendrocyte glycoprotein; OPC, oligodendrocyte precursor cells; PID, post-immunization day; PLP, myelin proteolipid protein; PTX, Pertussis toxin; TMEV, Theiler’s murine encephalomyelitis virus; W, week.

## Toxin-induced central nervous system demyelination

One robust way to model demyelination and remyelination is via the use of cuprizone induced-demyelination. Cuprizone (biscyclohexanone oxaldihydrazone) is a copper-chelating chemical that is orally administered to rodents to induce oligodendrocyte death, glial cell activation, and demyelination via mitochondrial dysfunction (Matsushima and Morell, 2001). Cuprizone’s presumed mechanism of action is through mitochondrial injury driven by the suppression of complex IV of the electron transport chain and oxidative stress due to the increased formation of reactive oxygen species (ROS) and subsequent DNA damage. These mitochondrial-linked impairments reduce the energy supply for the cell, leading to cell starvation, injury, and death (Faizi et al., 2016).

The cuprizone model can be segmented into two stages: CNS demyelination in the corpus callosum and other parts of the cerebrum accompanied by innate immune activation followed by extensive remyelination of the denuded axons upon cessation of cuprizone (Toomey et al., 2021). In the acute stage, demyelination occurs within 4-6 weeks of oral cuprizone exposure (Zhan et al., 2020). Demyelination appears to be directly linked with mature oligodendrocyte death, which starts occurring within 1–2 weeks of toxin exposure. Cuprizone-induced innate immune activation within the CNS follows as reactive microglia and neurotoxic astrocyte phenotypes become more prominent.

Following the demyelination that occurs upon acute cuprizone ingestion, oligodendrocyte precursor cells (OPCs) begin to proliferate and migrate to the areas of demyelination, even while the animals are ingesting cuprizone since these non-myelinating cells are much less metabolically active and can survive the mitochondrial toxicity. However, successful remyelination is dependent on cessation of cuprizone to allow OPCs to differentiate into mature myelinating oligodendrocytes. OPCs have been shown to be the major source of remyelination in this model. In the acute cuprizone model, the toxin is discontinued at 3–6 weeks allowing remyelination to occur in the ensuing 2–4 weeks. Cuprizone diet triggered extensive corpus callosum (CC) demyelination, reduced axon conduction, and resulted in loss of axon structural integrity including nodes of Ranvier. Indeed, there is a window of opportunity for axonal remyelination following cuprizone withdrawal. Demyelination longer than 1.5 weeks can cause axonal injury that limits successful remyelination of axons to normal levels by oligodendrocytes (Crawford et al., 2009). In the chronic cuprizone model, the toxin is administered for 8–12 weeks, and axonal damages ensues limiting the potential for complete remyelination. The corpus callosum also thins in the chronic model as a sequela of axonal injury that occurs in MS. Although acute cuprizone treatment allows for more rapid remyelination, chronic cuprizone treatment still results in myelin repair of the surviving axons (Kipp et al., 2009; Lindner et al., 2009).

Other toxin-based methods to reproduce demyelination and remyelination as may occur in MS pathology include lysolecithin (lysophosphatidylcholine—LPC) and diphtheria toxin administration. LPC non-specifically binds to myelin and disrupts the lipid architecture. LPC has been shown to rapidly integrate into the membranes of cells and similarly cause deformation and subsequent permeabilization of these cells (Plemel et al., 2017). In the diphtheria toxin (DT) model, the diphtheria fragment A gene (DTA) can be conditionally expressed in oligodendrocytes, leading to oligodendrocyte death and CNS demyelination (Popko, 2010). Alternatively, transgenic mice have been made with the diphtheria toxin receptor expressed under an oligodendrocyte specific promoter and upon deliver of diphtheria toxin rapid demyelination ensues (Brockschnieder et al., 2006; Popko, 2010; Traka et al., 2010; Pohl et al., 2011; Plemel et al., 2017).

There are many beneficial aspects of utilizing toxins to model the myelination processes that occur in MS. Acute and chronic demyelination with associated CNS atrophy can be modeled using cuprizone, LPC, and diphtheria toxin and have similarities to aspects of human MS pathology (Peterson et al., 2001; Buch et al., 2005; Kutzelnigg et al., 2005; Traka et al., 2010; Goldberg et al., 2015; Wagenknecht et al., 2016; Pang et al., 2022). Axonal damage in demyelinated lesions along with minor peripheral immune infiltration are similarly aligned with progressive MS (Höflich et al., 2016; Singh et al., 2017; Machado-Santos et al., 2018). Axons are more preserved in the LPC model as opposed to prolonged cuprizone exposure (Kirk et al., 2003; Berghoff et al., 2017; Filippi et al., 2018; Chrzanowski et al., 2019).

Despite the advantages of using these toxins to induce demyelinating pathology, there are various limitations to consider when attempting to model MS. Given that cuprizone is a toxin that poisons mitochondria activity and induces a degenerative loss in the CNS, the adaptive immune system characteristics of MS are lacking (Baxi et al., 2015). Indeed, cuprizone-induced demyelination is not lymphocyte-dependent and occurs even in lymphocyte-deficient recombination activating gene (*Rag*) knockout mice (Hiremath et al., 2008). Additionally, susceptibility to cuprizone pathology can vary based on animal weight and genetic background, cuprizone consumption and formulation means of cuprizone delivery, and animal age (Carey and Freeman, 1983; Zatta et al., 2005; Irvine and Blakemore, 2006; Taylor et al., 2010; Steelman et al., 2012; Hochstrasser et al., 2017; Yu et al., 2017; Leopold et al., 2019). In the following sections, we will focus on inflammatory immune response in toxin-based demyelination models and review several models that have incorporated an induced immune response to further our understanding of potential mechanisms of remyelination failure in MS.

## Toxin-based demyelination and inflammatory immune response

Many studies show the autoimmune origin of MS, in which autoreactive T cells drive multifocal and heterogeneous inflammatory demyelination (Rodríguez Murúa et al., 2022). Some findings suggest that brain-intrinsic pathologies such as primary apoptosis of oligodendrocytes trigger the recruitment of peripheral immune cells into the CNS. Based on these observations, two models have been proposed to describe the etiology of MS, the “outside-in” and the “inside-out” models (Titus et al., 2020). The outside-in paradigm suggests that immune cells are primarily activated in the periphery and attack the CNS, while inside-out implicates a primary CNS cytotoxic process such as oligodendrocyte death that triggers secondary autoimmune reactions against myelin antigens (Stys et al., 2012). The cuprizone model is considered an appropriate approach to test the “inside-out” hypothesis since cuprizone toxicity is lymphocyte-independent and primarily caused by oxidative stress and mitochondrial dysfunction of oligodendrocytes (recently reviewed by Zirngibl et al., 2022).

Some studies tested whether cuprizone-induced demyelination could activate the adaptive immune response, causing T cell infiltration into the CNS. However, there are controversial observations regarding the impact of cuprizone on inflammatory demyelination models. Earlier studies suggest that acute cuprizone intoxication does not induce a blood–brain barrier (BBB) permeability (Bakker and Ludwin, 1987; Kondo et al., 1987; McMahon et al., 2002) and T cell infiltration to the CNS (Matsushima and Morell, 2001; Remington et al., 2007; Hiremath et al., 2008). Even after pertussis toxin administration to breach the BBB, T cell infiltration is not detectable in the CNS (Sen et al., 2019). In contrast, other studies show that BBB integrity is compromised in mice and precedes the demyelination and glial activation observed in cuprizone treatment (Berghoff et al., 2017; Shelestak et al., 2020). Cuprizone-induced demyelination may trigger the recruitment of specific peripheral immune cells, particularly CD8 + lymphocytes, which predominantly display a cytotoxic phenotype with high proliferation rates and cytotoxic granule expression. This finding indicates an antigenic and pro-inflammatory milieu in the CNS of cuprizone-treated mice (Kaddatz et al., 2021).

It is unclear whether infiltrating T cells are activated against myelin antigens in the cuprizone model. Although myelin is found in the cervical lymph nodes following cuprizone-treatment, it seems not to be sufficient to trigger an immune-mediated CNS inflammation (Scheld et al., 2016). It is shown that the numbers of inflammatory effector T cells (Th_1_ and Th_17_ cells) are unchanged in peripheral lymphoid organs and adoptive transfer of splenocytes from cuprizone-treated animals to recipient mice does not result in CNS autoimmunity (Scheld et al., 2016). Similar results were reported by Locatelli et al. using oDTR model, in which mature oligodendrocyte ablation is induced by the administration of diphtheria toxin. Despite the presence of myelin antigens in the CNS-draining lymph nodes, T cells are not primed against myelin antigens even after pertussis toxin administration as an enhancer of CNS inflammation (Locatelli et al., 2012). These findings suggest that oligodendrocyte death would not be enough to trigger an adaptive immune response and trafficking of the peripheral immune cells into the CNS. Interestingly, another study using a diphtheria toxin (DTA) mouse model suggests that myelin degeneration can cause immune-mediated neuroinflammation and tissue damage at the later stage (Traka et al., 2016). Oligodendrocyte ablation using diphtheria toxin in Plp1-CreER(T);ROSA26-eGFP-DTA (DTA) mouse model induces a fatal late-onset disease (approximately 30 weeks after recovering from oligodendrocyte loss and demyelination), associated with extensive demyelination, T cell infiltration to the CNS, and axonal loss. Myelin oligodendrocyte glycoprotein (MOG)-specific T cells were found in lymphoid organs that could induce EAE in naive recipients upon adoptive transfer. Taken together, this study indicates that oligodendrocyte death in the chronic stage would be sufficient to trigger an adaptive autoimmune response against myelin (Traka et al., 2016).

T cell recruitment was also reported when cuprizone intoxication was combined with a non-specific immune stimulus such as Complete Freund’s Adjuvant (CFA) and pertussis toxin (Caprariello et al., 2018). In this model, known as cuprizone autoimmune encephalitis (CAE), mild cuprizone intoxication (only 2 weeks) is followed by a non-specific activation of immune cells using CFA and pertussis toxin (Caprariello et al., 2018). Substantial astrogliosis and infiltration of leukocytes including T cells and macrophages are observed in CAE lesions, indicating secondary inflammatory demyelination like active MS lesions. This finding suggests that subclinical myelin injury may trigger the neuroinflammation that subsequently results in oligodendrocyte loss and demyelination. This finding provides a rationale for the inside-out model of MS pathogenesis.

## Demyelination combined with central nervous system autoimmunity

Toxin-based models largely lack the inflammatory microenvironment that exists in the MS lesions. In the EAE model, the infiltration of inflammatory immune cells causes severe demyelination and axonal degeneration, limiting the ability of the model to test the novel remyelination strategies (Soulika et al., 2009). In the cuprizone model, however, a robust demyelination and remyelination occur in predictable time frame and regions of the CNS but the endogenous repair is very fast (within 2–3 weeks), providing a limited period to test the drugs that accelerate the spontaneous repair process (Vega-Riquer et al., 2019). For these reasons, combinational models have been developed to enable the study of remyelination in the context of ongoing inflammation (Baxi et al., 2015; Rüther et al., 2017).

Several studies reported that cuprizone treatment can modulate the course and the severity of EAE, induced by active immunization with myelin antigens (MOG or PLP) or Theiler’s murine encephalomyelitis virus (TMEV) (Emerson et al., 2001; Maña et al., 2009; Herder et al., 2012). It is shown that cuprizone intoxication generates adaptive immune response against several myelin components that are tolerogenic and/or decrease CD4+/CD8+ ratio, thereby impedes progression of EAE (Emerson et al., 2001; Maña et al., 2009). Cuprizone has also shown to be immunosuppressive and causes the atrophy of peripheral lymphoid organs such as the thymus or spleen (Solti et al., 2015; Sen et al., 2019). In contrast, there are studies that identified a comparable severity of EAE clinical symptoms in mice with or without cuprizone intoxication.

Recent studies describe a model combining cuprizone demyelination with the classic active EAE (Cup/EAE). In this model, inflammatory lesions are detected at various forebrain areas, including white matter, cortical, and subcortical gray matter (Scheld et al., 2016; Rüther et al., 2017). The neurodegenerative process was induced by cuprizone feeding of adult mice for 3 weeks, followed by a period on normal chow (Scheld et al., 2016). Subsequent immunization with MOG 35–55 peptide induces myelin autoreactive T cells in the periphery, resulted in massive recruitment of immune cell including encephalitogenic T cells together with monocytes into the affected forebrain. In addition, mice fed cuprizone before adoptive T-cell transfer showed a higher number of perivascular infiltrates compared with animals without previous cuprizone feeding (Scheld et al., 2016; Rüther et al., 2017). This study clearly illustrates the significance of brain-intrinsic degenerative cascades for immune cell recruitment and lesion formation. Similarly, Locatelli et al. tested whether oligodendrocyte death interfered with EAE development using oDTR mice. The EAE clinical scores and disease incidence were found comparable in all of the mice and the level of activated inflammatory infiltrates were similar at the onset (Locatelli et al., 2012). These findings suggest that cuprizone itself but not oligodendrocyte death ameliorates the course of EAE.

To overcome the limitation of cuprizone in the active EAE model, our group developed a adoptive transfer cuprizone model (ATCup) by combining cuprizone and adoptive transfer of activated myelin-specific CD4+ T cells. Mice are fed cuprizone for 4 weeks to induce demyelination and then adoptively transferred with myelin reactive CD4+ T cells that have been polarized to the Th17 profile *in vitro* (Baxi et al., 2015). We found IFNγ/IL-17 dual producing CD4 + T cells in the brain of cuprizone-fed mice following adoptive transfer, resulting in the significant inhibition of spontaneous remyelination. While in this system, the ensuing CNS inflammatory response inhibits endogenous remyelination, axons are preserved (unlike in the spinal cord of EAE animals). This preservation of axons allows for the capacity to examine the efficacy of drugs on OPC differentiation in a system where remyelination typically fails, similar to what may happen in MS plaques.

To better understand the mechanisms by which myelin reactive T cells inhibit remyelination, we performed AT-Cup in a lineage tracing OPC reporter mice (PDGFRα-CreER x Rosa26-YFP), in which we fate mapped OPCs in the regular cuprizone model (Baxi et al., 2017). In this model, OPC-specific Cre recombination occurs upon administration of 4-hydroxytamoxifen (4-HT) during the 3rd week of cuprizone and OPCs become positive for yellow fluorescent protein (YFP), a heritable trait that will remain even as they divide or differentiate into oligodendrocytes. We assessed the differentiation of YFP^+^ OPCs during remyelination and after adoptive transfer of activated T cells and found that inflammatory T cells inhibit the differentiation of OPCs into mature oligodendrocytes in the corpus callosum (Kirby et al., 2019).

Taken together, the combinational inflammatory remyelination models, Cup/EAE and ATCup, could be useful for testing remyelination therapies for MS in the context of active inflammation. In this setting, T cells produce inflammatory cytokines (IFNγ and IL-17) that suppress spontaneous remyelination. In addition, microglia and astrocytes respond to T cell cytokines and are polarized toward a reactive phenotype that further suppress remyelination (Gharagozloo et al., 2022; Kunkl et al., 2022).

## Demyelination in the genetic models of inflammatory central nervous system

One of the inflammatory factors that has been implicated in impaired remyelination in MS lesion is IFNγ, mainly produced by activated T cells and natural killer cells. To examine the molecular mechanisms of IFNγ-induced oligodendrocyte injury, the Popko lab used transgenic mice that allow temporally controlled expression of IFNγ in the CNS using the tetracycline controllable system (Lin et al., 2006). These animals exploit the transcriptional regulatory region of the glial fibrillary acidic protein (GFAP) gene to drive a tetracycline-controlled transcriptional activation (tTa) system’s expression in astrocytes as an ectopic source of IFNγ in the CNS. *GFAP/tTA* mice were crossed with *TRE/IFN*γ mice to produce *GFAP/tTA*;*TRE/IFN*γ double transgenic animals (Lin et al., 2004), in which mice begin expressing IFNγ in the forebrain after 2 weeks of doxycycline removal. It is shown that ectopic expression of IFNγ in the CNS after cuprizone-induced demyelinating results in reduced oligodendroglia maturation and impaired remyelination (Lin et al., 2006).

Mechanistically, IFNγ can directly affect oligodendrocytes, inducing endoplasmic reticulum (ER) stress response that leads to the apoptotic cell death (Lin et al., 2006). Moreover, we found that IFNγ polarizes OPC to an immune phenotype (iOPCs), in which OPC express immunoproteasome subunit and MHC class I, allowing cross-presentation of antigen to cytotoxic CD8 T cells and resulting in OPC death (Kirby et al., 2019). IFNγ may also have a secondary effect on oligodendrocytes, possibly through the activation of microglia and polarizing astrocytes toward a reactive/neurotoxic phenotype (Liddelow et al., 2017). Activated microglia produce inflammatory mediators including interleukin-1α (IL-1α), tumor necrosis factor (TNF) and complement component 1q (C1q) that generate reactive astrocytes. Toxic lipoparticles containing saturated lipids (APOE and APOJ) are secreted by neurotoxic astrocytes that mediate oligodendrocytes death (Guttenplan et al., 2021). We have recently examined the functional effect of neurotoxic astrocyte conditioned media (ACM) on OPC using a myelin basic protein (MBP) reporter line. Our data show that reactive astrocytes are not cytotoxic to the OPCs but robustly inhibit their maturation to oligodendrocytes, thereby limiting their remyelination capacity (Smith et al., 2022).

## Concluding remarks

There are many model systems to capture specific facets of MS pathophysiology. However, it is exceedingly difficult to recapitulate most features of MS within the same model given the multifaceted, complex neuroimmunological and neurodegenerative pathology that occurs. Nevertheless, several MS therapeutics have been clinically developed in the EAE model that have shown proven benefit in MS. However, remyelination therapy for MS remains challenging since many remyelinating drugs have shown promise pre-clinically but have had minimal benefit in clinical trials. The translational gap between animal models and clinical trials can be bridged by developing combinational models that better recapitulate MS pathology, incorporating the influence of inflammatory molecules that inhibit remyelination within the lesion. There is much excitement within the field as it progresses with the use of combinatorial model systems that incorporate the main hallmarks of MS such as remyelination and autoimmune immunopathology, innate immune -mediated inflammation, and neurodegeneration. Understanding the complex mechanisms underlying impaired remyelination in MS will provide us with more targeted and effective therapeutic approaches that promote remyelination and halt MS disease progression.

## Author contributions

All authors designed the format, wrote the entire manuscript, and read and approved the manuscript.
